# Substantial Improvement of High Temperature Strength of New-Generation Nano-Oxide-Strengthened Alloys by Addition of Metallic Yttrium

**DOI:** 10.3390/ma15020504

**Published:** 2022-01-10

**Authors:** Jiří Svoboda, Petr Bořil, Jakub Holzer, Natália Luptáková, Milan Jarý, Bohuslav Mašek, Petr Dymáček

**Affiliations:** 1 Institute of Physics of Materials, Czech Academy of Sciences, Žižkova 22, 616 62 Brno, Czech Republic; boril@ipm.cz (P.B.); holzer@ipm.cz (J.H.); luptakova@ipm.cz (N.L.); jary@ipm.cz (M.J.); pdymacek@ipm.cz (P.D.); 2 Faculty of Electrical Engineering, University of West Bohemia, Univerzitni 8, 306 14 Pilsen, Czech Republic; masekb@email.cz

**Keywords:** mechanical alloying (MA), oxide dispersion strengthened (ODS) alloy, powder hot consolidation by rolling, tensile strength at 1100 °C, alloying with metallic yttrium

## Abstract

Oxide-dispersion-strengthened (ODS) Fe-Al-Y_2_O_3_-based alloys (denoted as FeAlOY) containing 5 vol. % of nano-oxides have a potential to become top oxidation and creep-resistant alloys for applications at temperatures of 1100–1300 °C. Oxide dispersoids cause nearly perfect strengthening of grains; thus, grain boundaries with limited cohesive strength become the weak link in FeAlOY in this temperature range. One of the possibilities for significantly improving the strength of FeAlOY is alloying with appropriate elements and increasing the cohesive strength of grain boundaries. Nearly 20 metallic elements have been tested with the aim to increase cohesive strength in the frame of preliminary tests. A positive influence is revealed for Al, Cr, and Y, whereby the influence of Y is enormous (addition of 1% of metallic Y increases strength by a factor of 2), as it is presented in this paper.

## 1. Introduction

The application of advanced materials with excellent oxidation and high-temperature creep resistance enables an increase in operating temperature of heat engines and, thus, an increase in efficiency with a positive impact on the environment. Top-level metallic materials with the best creep performance are represented by Ni-based superalloy single crystals [1] that are applicable up to 1100 °C and by oxide-dispersion-strengthened (ODS) ferritic alloys [2,3] applicable up to 1300 °C. In both cases, excellent creep resistance is achieved by the Orowan mechanism due to the dispersion of particles effectively prohibiting dislocation motion at high temperatures.

Classical ferritic ODS alloys are strengthened by 0.5% volume fraction of very stable Y-based nano-oxides typically of 5–20 nm in size [4]. The ferritic matrix of classical ODS alloys allows dissolving a rather high amount of Al (up to 10%, note: wt. % is used in all notation), which stabilizes bcc lattice and excludes transformation of ferrite to austenite.

Classical ODS alloys are usually produced in two steps. In the first step, a homogeneous powder consisting of a matrix with dispersed nano-sized Y_2_O_3_ is produced by mechanical alloying (MA). In the second step, the powder is consolidated via hot extrusion, hot isostatic pressing, spark plasma sintering, or via their combinations [5,6,7,8,9,10,11]. Auger et al. [12] applied hot cross rolling to improve the resistance of 14YWT ODS alloys against radiation. Zhang et al. [13] used hot rolling to improve the strength of Fe–9Cr-0.06C-1.5W-0.5Ti-0.18Si-0.35Y_2_O_3_ ODS alloys after hot consolidation. Kumar et al. [14] investigated mechanical properties of Fe-18Cr-2W-0.2Ti-xY_2_O_3_ alloys consolidated from MA powder by forging. Zhou et al. [15] applied combinations of forging, cold rolling, hot rolling, and annealing to ODS310 alloys. However, incomplete secondary recrystallization results in inhomogeneous bimodal grain size distribution causing brittle cracking along grain boundaries in ODS alloys [16]. All these methods of consolidation and subsequent thermomechanical treatment are complicated and expensive.

A new-generation of ODS Fe-Al-Y_2_O_3_-based alloys (denoted as FeAlOY) containing a high-volume fraction of oxides of about 5%, which is one order of magnitude higher than in classical ODS alloys [3,17], has been developed by the authors. The consolidation by hot rolling results in ultra-fine-grained (UFG) microstructure due to dynamic recrystallization. Such a material is hard and brittle at room temperature and soft and ductile at 1100–1300 °C. The coarse-grained microstructure obtained by full secondary recrystallization is the key factor for acquiring excellent creep strength. However, several ODS alloys studied in the available literature do not meet this requirement (see, e.g., [16]). Our recent experiments clearly indicate that UFG microstructure after unsuccessful secondary recrystallization or incomplete secondary recrystallization in ODS alloys causes its creep strength to reach only a fraction of the creep strength of completely recrystallized ODS alloys. Furthermore, expensive ODS alloys are often studied and applied at loading conditions for which much cheaper and better substitutes exist. One could recommend limiting studies and applications of ODS alloys to temperatures and loading conditions at which the properties of ODS alloys are really exceptional.

Recent tensile testing at low strain rates (10^−6^ s^−1^) and creep tests at 1100 °C have revealed that strengthening the matrix by nano-oxide dispersion is very effective due to the Orowan mechanism. Under loading corresponding to 60–70% of the strength determined by the tensile test, the creep strain rate is practically unmeasurable (<10^−9^ s^−1^), and the time to fracture reaches several thousands of hours. Then, the grain boundaries with limited cohesive strength become weak links in FeAlOY at 1100–1300 °C. One of the possibilities for significantly improving the strength of the FeAlOY is alloying appropriate elements and, thus, increasing the cohesive strength of grain boundaries. Nearly 20 metallic elements have been tested with the aim to increase cohesive strength within the preliminary study (unpublished results). The study reveals that the cohesive strength of grain boundaries increases with the content of Al and, thus, the content of Al in FeAlOY is fixed to 10%, which is the solubility limit of Al in ferrite at low temperatures. Such a high content of Al ensures excellent oxidation resistance [18] as well as a significant decrease in density to 6.5 g/cm^3^. The role of alloying with Cr seems to be at least neutral and may open quite interesting possibilities. Thus, it must be investigated in combination with other alloying elements in more detail in the near future.

Originally, the addition of metallic Y to FeAlOY has been motivated by an improvement in the stability of oxide dispersion against coarsening because pure Y_2_O_3_ oxide is chemically more stable than mixed (Al,Y)_2_O_3_ oxides; therefore, it is more resistant against coarsening [3]. As a certain amount of O is introduced into FeAlOY by using powders naturally oxidized at the surface for FeAlOY production, it is necessary to compensate O by the addition of metallic Y to gain pure Y_2_O_3_ nanodispersoid. This is why a series of experiments on FeAlOY with different amounts of added metallic Y is performed. These tests also include tensile tests at strain rates 10^−6^ s^−1^ and 1100 °C. The study provides unprecedented results, which are presented in this paper.

## 2. Materials and Methods

Chemically homogeneous Fe-10Al-4Y_2_O_3_ and Fe-10Al-4Cr-4Y_2_O_3_ powders with variable amounts of added metallic Y in the range of 0 to 1.75% are prepared by MA from Fe, Al, and Y_2_O_3_ powders and from Cr and Y granulates of 99.9% purity. A vacuum-tight milling container has the volume of 22 dm^3^ and a diameter of 400 mm; it is made from low alloyed steel and is filled with 100 martensitic bearing balls (Fe-1Cr-1C) of 40 mm diameter. A total of 1.5 kg of the powder is mechanically alloyed in a vacuum by rotation of the milling container along the horizontal axis (70 rpm). After two weeks of MA, the homogeneous powder particles consist of a solid solution with a large amount of defects, such as dislocations and vacancies [3]. More detailed powder characterization can be found in [19]. The MA powder is subsequently hot consolidated by rolling. The powder is canned in an evacuated 20/1 mm steel tube and hot consolidated by rolling in three steps to a thickness of 8 mm at 870 °C, to a thickness of 5 mm at 900 °C, and to a thickness of 3.25 mm at 900 °C. Then, the hot rolled sheet is stripped from the can and annealed at 1200 °C/4 h to provoke secondary recrystallization. The oxides nucleate and grow during hot rolling up to a size of 5 nm; then, they coarsen to 20–30 nm during annealing, provoking secondary recrystallization [20].

Flat specimens with a gauge length of 25 mm and cross section of 2.5 mm × 3.5 mm are cut by a precise electrical discharge machine from rolled sheets and grinded to P400. Tensile tests are performed by using the Zwick/Roell KAPPA DS 50 kN creep test system (Zwick/Roell, Fürstenfeld, Austria) with a furnace up to 1200 °C. Microstructures are observed by using SEM (scanning electron microscope/microscopy) Tescan Lyra 3 XMU FEG/SEMxFIB (Tescan, Brno, Czech Republic) equipped with a X-Max80 EDS detector for X-ray microanalysis and a symmetry electron backscatter diffraction (EBSD) detector with an Aztec control system (Oxford Instruments, Abingdon, UK). The X-ray diffraction (XRD) measurement is performed on a PanAnalytical Empyrean automated diffractometer in Bragg–Brentano configuration (Malvern Pananalytical Ltd., Malvern, United Kingdom), which uses a 1D detector and a cobalt lamp (λ-Kα_1,2_ = 1.788965, 1.792850 Å, acceleration voltage of 30 kV and current of 20 mA) with a β filter. The specimens are measured in continuous mode with steps of 0.04 2θ° from 10 to 130 degree range while they rotate. Quality and quantity are interpreted by the HighScorePlus 4.0 software, utilizing the Rietveld method and the ICSD database (2017).

## 3. Tensile Testing

**Figure 1:**
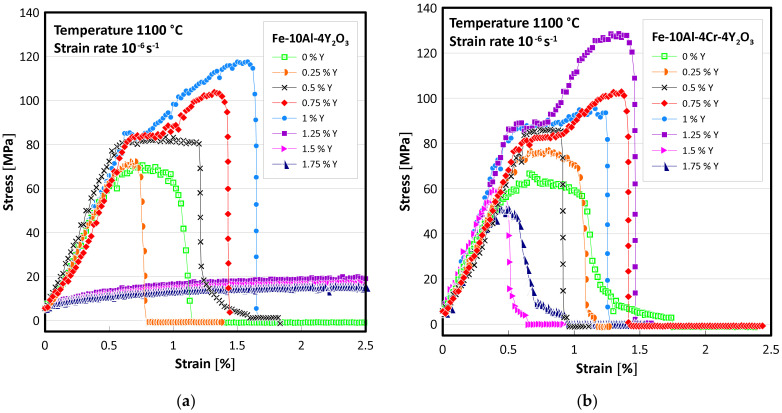
The typical stress-strain curves of (**a**) Fe-10Al-4Y_2_O_3_ and (**b**) Fe-10Al-4Cr-4Y_2_O_3_ ODS alloys with addition of different amounts of Y.

**Figure 2:**
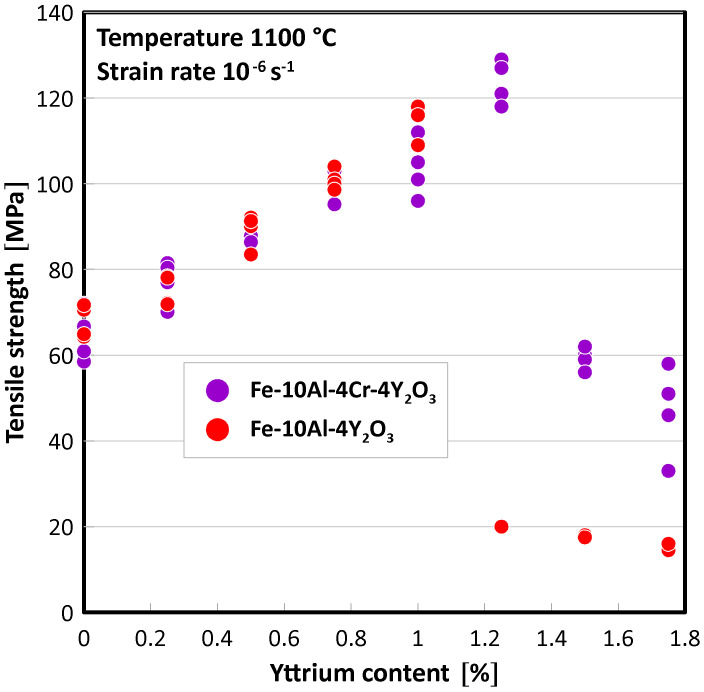
Measured tensile strengths of all specimens of Fe-10Al-4Y_2_O_3_ and Fe-10Al-4Cr-4Y_2_O_3_ ODS alloys with addition of different amounts of Y. Several values coincide, and they are not visible.

Four specimens of each of the Fe-10Al-4Y_2_O_3_ and Fe-10Al-4Cr-4Y_2_O_3_ ODS alloys with additions of 0, 0.25, 0.5, 0.75, 1, 1.25, 1.5, and 1.75% Y are prepared and mechanically tested. Tensile tests at a strain rate of 10^−6^ s^−1^ and a temperature of 1100 °C are performed on all 64 prepared specimens. The typical stress strain curves are presented in Figure 1, and the results of all tensile tests are summarized in Figure 2. From Figure 1 and Figure 2, it is evident that strength increases with increasing Y content in both Fe-10Al-4Y_2_O_3_ and Fe-10Al-4Cr-4Y_2_O_3_ ODS alloys up to a certain limit; it then drops to much lower values. In the case of high strength, low ductility is observed, which indicates the existence of a significant portion of intergranular brittle fracture, and this can be explained by grain boundary decohesion. Thus, the cohesion strength of grain boundaries seems to be the weakest link in the specimens, and the strength of the specimens can be considered as a measure of the cohesive strength of the grain boundaries, provided that the grain microstructure of the specimens is similar. In order to judge this assumption, a relevant microstructure characterization by SEM is performed.

## 4. Microstructure Characterization

**Figure 3:**
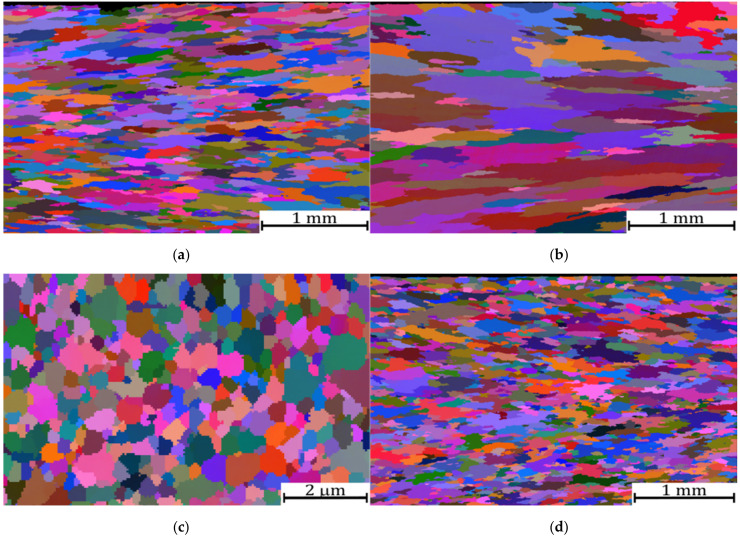

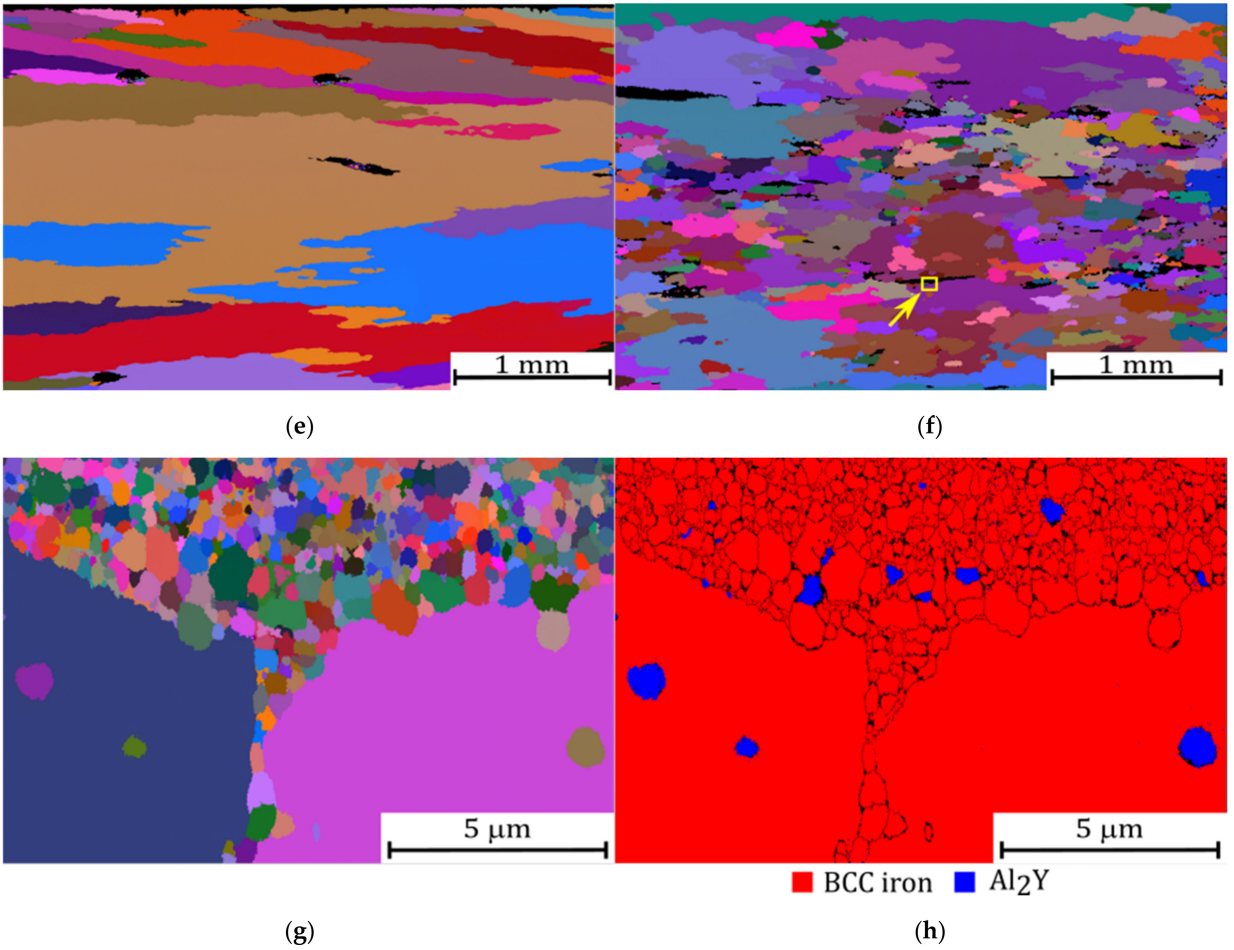
Euler color EBSD maps of grain microstructure for Fe-10Al-4Y_2_O_3_ ODS alloys with (**a**) 0, (**b**) 1, and (**c**) 1.75% Y and for Fe-10Al-4Cr-4Y_2_O_3_ ODS alloys with (**d**) 0, (**e**) 1.25, and (**f**,**g**) 1.75% Y. (**h**) is the phase map corresponding to (**g**) indicating precipitation of Al_2_Y (blue phase). The (**g**) and (**h**) maps correspond to a yellow rectangle in map (**f**).

**Figure 4:**
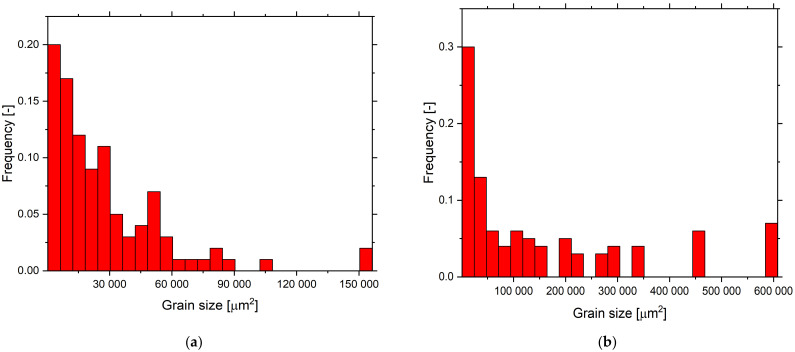

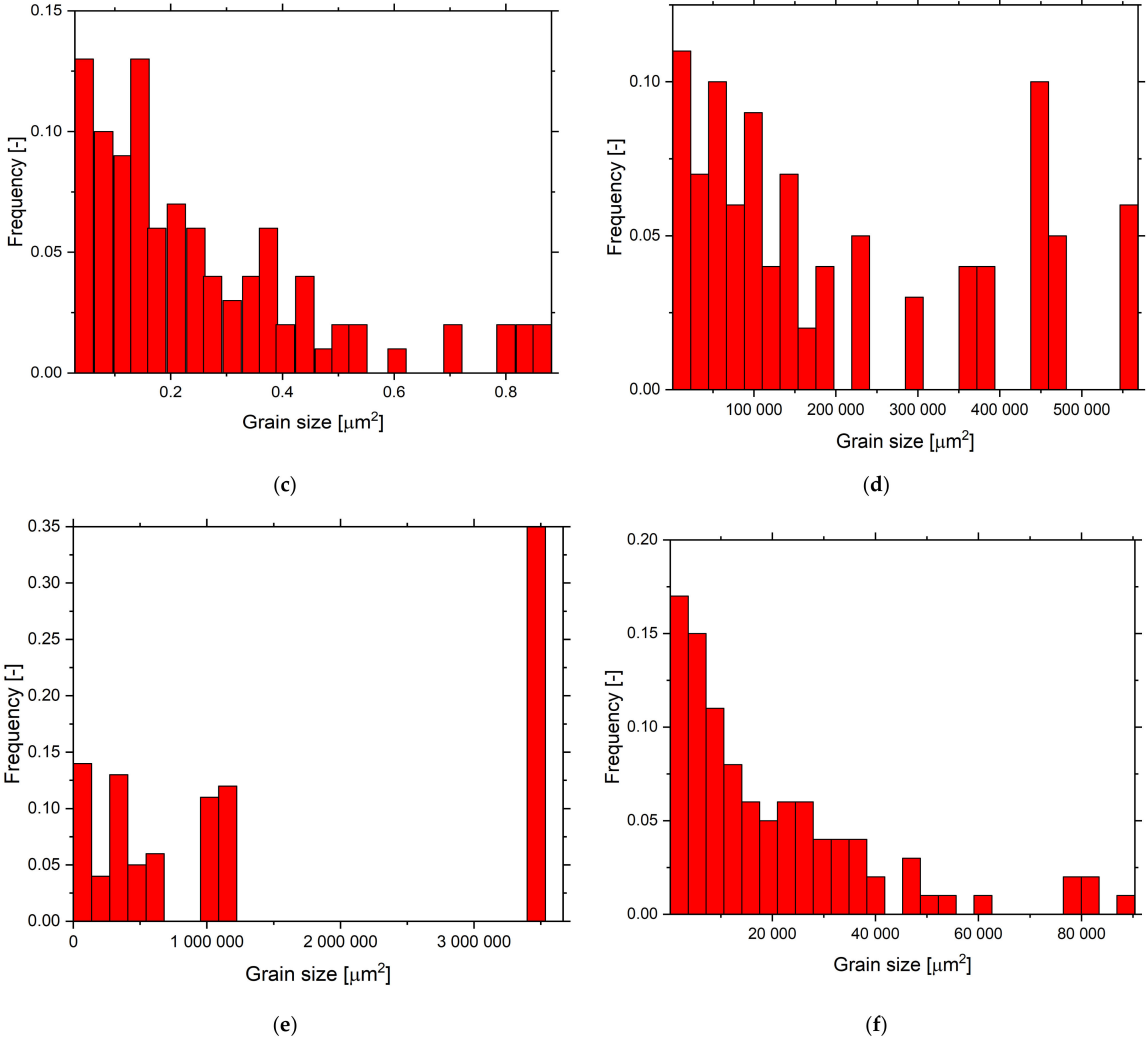
Histograms of grain areas for Fe-10Al-4Y_2_O_3_ ODS alloys and aspect ratios (numbers in brackets) with (**a**) 0% Y (2.7), (**b**) 1% Y (5.7), and (**c**) 1.75% Y (1.7) and for Fe-10Al-4Cr-4Y_2_O_3_ ODS alloys with (**d**) 0% Y (3.6), (**e**) 1.25% Y (6.7), and (**f**) 1.75% Y (2.5).

Grain microstructures are characterized by EBSD for Fe-10Al-4Y_2_O_3_ ODS alloys with 0, 1, and 1.75% Y and for Fe-10Al-4Cr-4Y_2_O_3_ ODS alloys with 0, 1.25, and 1.75% Y, and the results are presented in Figure 3. Generally, the grains after secondary recrystallization are pancake-shaped and lay on the rolling plane. The Euler color EBSD maps in Figure 3 are taken from the body of the specimen; they cover its entire thickness, while the x-axis of the map is parallel to the rolling direction, and the y-axis is normal to the rolling plane. Figure 3a–c correspond to 0%, 1%, and 1.75% of Y in Fe-10Al-4Y_2_O_3_ ODS alloys, and Figure 3d–h correspond to 0%, 1.25%, and 1.75% of Y in Fe-10Al-4Cr-4Y_2_O_3_ ODS alloys. The grain microstructures for 0% of Y (Figure 3a,d) consist of grains of a mean size of about 200 µm elongated in the rolling direction with an aspect ratio of about 2. Figure 3b,e correspond to 1% Y in Fe-10Al-4Y_2_O_3_ and 1.25% Y in Fe-10Al-4Cr-4Y_2_O_3_ ODS alloys, respectively, and they consist of very large grains of sizes that are over 1 mm elongated in the rolling direction with an aspect ratio over 2. One can also realize that the grains near the surface of the specimen are significantly finer, and there are sporadic regions without secondary recrystallization (see Figure 3e for 1.25% Y in the Fe-10Al-4Cr-4Y_2_O_3_ ODS alloy). Figure 3c depicts the Euler color EBSD map of the specimen with 1.75% Y in Fe-10Al-4Y_2_O_3_ in which no secondary recrystallization occurs during annealing and the grains are equiaxed with a mean size of about 500 nm. The Euler color EBSD map in Figure 3f corresponds to the specimen with 1.75% Y in Fe-10Al-4Cr-4Y_2_O_3_, in which the grains are not fully recrystallized and exhibit prevailing coarse-grained microstructure of sizes between 0.2 and 2 mm with islands of fine equiaxed grains with a mean size of about 500 nm (see Figure 3f,g). Moreover, the phase map reveals precipitation of Al_2_Y shown in Figure 3h, which is due to the excess of Y, which cannot be dissolved in the matrix. Histograms of grain areas for Fe-10Al-4Y_2_O_3_ ODS alloys with (a) 0, (b) 1, and (c) 1.75% Y and for Fe-10Al-4Cr-4Y_2_O_3_ ODS alloys with (d) 0, (e) 1.25, and (f) 1.75% Y and the values of mean aspect ratios of the grains are presented in Figure 4. It is necessary to note that although the ultra-fine-grained microstructure is equiaxed after hot rolling, it changes to rather large pancaked grains oriented in the rolling plane due to secondary recrystallization (see Figure 3a,b,d,e) and remains as slightly coarsened and equiaxed in unrecrystallized regions (see Figure 3c,g).

**Figure 5:**
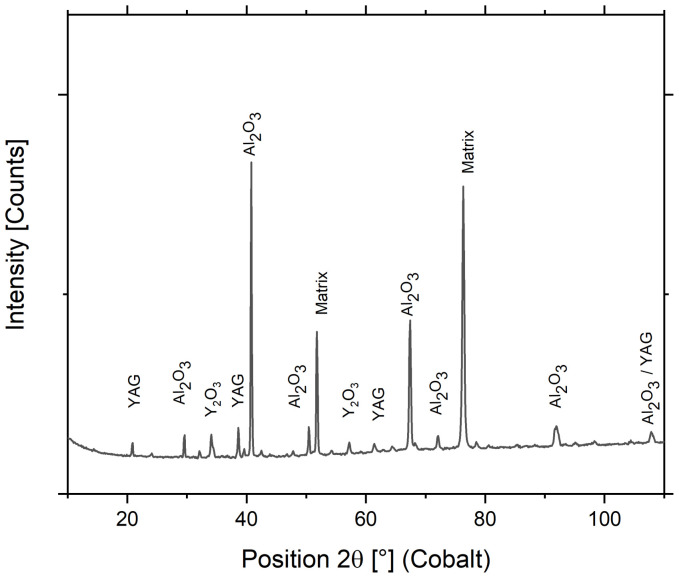
X-ray diffraction data measured on specimens’ surface after the tensile test in air atmosphere.

X-ray diffraction data are acquired in order to elucidate phase composition and lattice parameters of compounds present in the head of Fe-10Al-4Cr-4Y_2_O_3_ ODS specimen alloys with 1.25% of Y after the tensile test in the air atmosphere. The diffractogram displayed in Figure 5 shows labeled peaks of four phases (with space groups) corresponding to the bcc matrix (Im-3m) with Y_2_O_3_ (Ia-3) nanoprecipitates and to surface oxidized layer consisting of Al_2_O_3_ (R-3c) with precipitates of Y_3_Al_5_O_12_ YAG (Ia-d). It must be noted that the detected thin surface oxide layer ensures excellent long-term protection of the specimen against further oxidation. It must be noted that although Y_2_O_3_ nano-oxides are detected by X-ray diffraction, the evaluation of their volume fraction does not result in realistic values (5–6% as it follows from the inputs). In order to perform a significantly more precise analysis, methods based on transmission electron microscopy (TEM) must be utilized. In the case of the Fe-10Al-4Cr-4Y_2_O_3_ ODS alloy with 1.75% added Y, Al_2_Y (Fd-3m) precipitates are detected (see Figure 3h). In the case of Fe-10Al-4Y_2_O_3_ or Fe-10Al-4Cr-4Y_2_O_3_ ODS alloys with no or small amounts of added Y, one can expect precipitation of three types of nano-oxides—YAlO_3_ (YAP-Pnma), Y_4_Al_2_O_9_ (YAM-P2_1_/c), and Y_2_O_3_—which deserves a separate TEM study.

**Figure 6:**
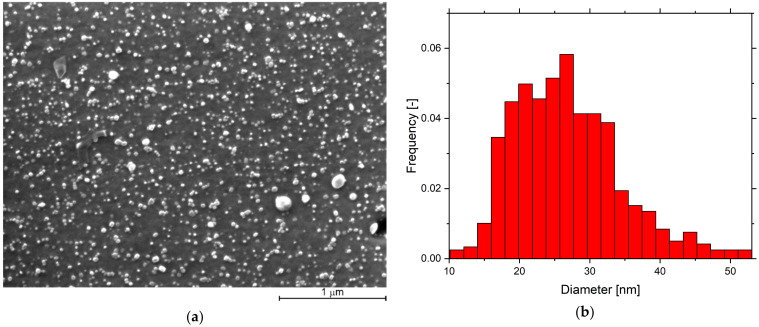
(**a**) The secondary electron SEM image of oxide dispersion in the section of the specimen in the grain interior of the recrystallized Fe-10Al-4Cr-4Y_2_O_3_ ODS alloy with 1.25% Y after final etching by the Vilella–Bain etchant and (**b**) its size distribution histogram.

**Figure 7:**
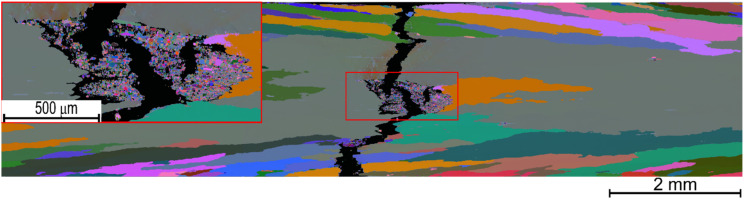
An Euler color EBSD map of Fe-10Al-4Cr-4Y_2_O_3_ ODS specimen with 1.25 Y showing coarse-grained microstructure and mixed trans/inter-crystalline fracture initiated in the soft unrecrystallized region.

An example of typical nano-oxide dispersion (practically independent of Y and Cr content) and its size distribution histogram in recrystallized grains is presented in Figure 6. The fracture mechanism of the Fe-10Al-4Cr-4Y_2_O_3_ ODS specimen alloy with 1.25 Y (with the highest strength) can be deduced from Figure 7. Here, it is clearly shown that the fracture is initiated in an unrecrystallized and much softer region in the central part of the specimen (some other unrecrystallized regions are also revealed in Figure 3e). This region can be considered as a defect, and one can expect that it could be removed by annealing at 1200 °C for a longer time than 4 h to fully complete secondary recrystallization.

## 5. Discussion

Y_2_O_3_ nano-oxides are extremely stable against coarsening and play a decisive role in strengthening FeAlOY up to temperatures of 1300 °C. Strengthening can be described by the Orowan theory, which allows the calculation of an increase of shear stress τ due to the presence of precipitates as τ=Gb/Δ, where G is the shear modulus, b is the length of the Burgers vector, and Δ is the mean distance between precipitates. By using the values of G≈40 GPa corresponding to 1100 °C (see [21] for the estimation of the temperature dependence of the elastic constants), $b=a\sqrt{3}/2\approx 0.25\;\mathrm{nm}$ with lattice constant a≈0.29 nm and Δ≈50 nm estimated from Figure 6 provide a value of τ≈200 MPa, which is well above the measured strengths presented in Figure 1. This supports our hypothesis that the strength of FeAlOY is determined by the cohesive strength of grain boundaries.

The present study clearly revealed that achieving an excellent tensile and creep strength at 1100 °C and higher temperatures is conditioned by complete secondary recrystallization resulting in very coarse grains. The results shown in Figure 2 and Figure 3 clearly indicate that the strengths of specimens exhibiting no or incomplete secondary recrystallization is significantly lower than that with complete or nearly complete secondary recrystallization. In the case of unrecrystallized grain microstructure, the strength is several times lower and ductility is very high at 1100 °C (see stress-strain curve for the highest amount of Y in Figure 1a). In the case of incomplete secondary recrystallization, both tensile strength and ductility are low (see stress-strain curve for highest amount of Y in Figure 1b), which indicates that the fracture is easily initialized in soft unrecrystallized regions, as demonstrated in Figure 5.

It is still not clear why an increase of Y content behind a certain limit blocks secondary recrystallization in the Fe-10Al-4Y_2_O_3_ ODS alloy and why it only slows down secondary recrystallization in the Fe-10Al-4Cr-4Y_2_O_3_ ODS alloy. Moreover, alloying with Cr increases the tendency of ODS alloys to complete secondary recrystallization. This is a promising finding that opens the door for further significant optimization of high temperature strength of the FeAlOY by varying the chemical composition.

The results in Figure 1 and Figure 2 show an unprecedented result: alloying by about 1% of a substitutional element (Y) increases the strength by a factor of 2 at 1100 °C. The increase in strength can be attributed to the following reasons:

i. Significant change of the grain microstructure and its morphology;
ii. Increase in cohesive strength of the grain boundary due to a change in its chemical composition (e.g., Y segregates grain boundaries and strengthens interatomic bonds);
iii. Change of chemical composition of the nano-oxides from mixed Al-Y oxides to pure Y oxides, which exhibit a improved wetting of the matrix; thus, the nano-oxides act as stronger clamps holding the grains together.

In any case, reason (i) is approved by this study; however, one cannot exclude that it is the only one. Reason (ii), which involves the segregation of elements at grain boundaries, can be identified by energy dispersive spectroscopy mapping in TEM. In order to test reason (iii), it is necessary to find appropriate hot consolidation conditions that provide similar grain structures in two FeAlOYs with different amounts of Y and, thus, possess different ratios of mixed Al-Y and pure Y oxides, which must be accurately characterized by TEM. The detailed analysis of this problem is a challenge for our team in the near future.

## 6. Conclusions

The conclusions can be summarized in the following items:

- Two batches of Fe-10Al-4Y_2_O_3_ and Fe-10Al-4Cr-4Y_2_O_3_ ODS alloys with variable amounts of added metallic Y in the range of 0 to 1.75% are prepared by mechanical alloying, hot consolidation by rolling, and annealing to provoke secondary recrystallization.
- The amount of added metallic Y substantially influences the strength of ODS alloys determined by tensile tests at 1100 °C and a strain rate of 10^−6^ s^−1^.
- Alloying by Y linearly increases the strength of ODS alloys up to a limit of about 1%, for which the strength increases by a factor of 2 compared to ODS alloys with no added Y. Beyond the limit, the strength drops by a factor of 6 in the case of the Fe-10Al-4Y_2_O_3_ ODS alloy and by a factor of 2 in the case of the Fe-10Al-4Cr-4Y_2_O_3_ ODS alloy.
- Three reasons are considered for explaining the increase in strength of ODS alloys by Y alloying: (i) by significant change of the grain microstructure and its morphology, (ii) by an increase in cohesive strength of the grain boundary due to change its chemical composition, and (iii) by a change of chemical composition of nano-oxides from mixed Al-Y oxides to pure Y oxides, which exhibit improved wetting of the matrix.
- Reason (i) (alloying by Y significantly influences the grain microstructure after annealing) explains qualitatively the behavior of the strength of ODS alloys. This, however, does not exclude the fact that reasons (ii) and (iii) do not play significant roles. Reasons (ii) and (iii) will be investigated in detail by our team in the near future.
